# Simultaneous Laryngeal and Mastoid Coccidioidomycosis: A Case Report and Literature Review

**DOI:** 10.7759/cureus.109217

**Published:** 2026-05-19

**Authors:** Gaurav A Jategaonkar, Austin R Swisher, Christopher F Saling, David G Lott, Nicholas L Deep

**Affiliations:** 1 Otolaryngology - Head and Neck Surgery, Mayo Clinic Alix School of Medicine, Phoenix, USA; 2 Otolaryngology - Head and Neck Surgery, Mayo Clinic Arizona, Phoenix, USA; 3 Infectious Diseases, Mayo Clinic Arizona, Phoenix, USA

**Keywords:** coccidioidomycosis ent manifestations, coccidioidomycosis head and neck, disseminated coccidioidomycosis, laryngeal coccidioidomycosis, mastoid coccidioidomycosis

## Abstract

A 72-year-old Caucasian woman from the Southwestern United States presented with chronic right ear pain, hearing loss, and progressive hoarseness. Prior treatments, including balloon sinuplasty and tympanostomy tube placement, failed to resolve her symptoms. Imaging revealed right mastoid opacification, while flexible laryngoscopy demonstrated erythematous and polypoid changes of the right larynx. Pathology from biopsies of both the middle ear and larynx revealed spherules consistent with *Coccidioides* infection. She was treated with systemic fluconazole and underwent tympanomastoidectomy with mastoid debridement. The patient’s postoperative course was uneventful, and she reported symptomatic improvement at follow-up. A multidisciplinary approach, including close coordination with infectious disease, guided long-term antifungal therapy. Coccidioidomycosis is a rare but important consideration in patients presenting with chronic head and neck symptoms, especially in endemic regions. This case highlights the potential for multifocal otolaryngologic involvement and underscores the need for a high index of suspicion in persistent or unexplained laryngeal and otologic disease. Diagnosis relies on biopsy and culture, and management typically requires both surgical and prolonged antifungal therapy. Multidisciplinary care is essential to optimize outcomes, particularly in cases involving bony structures or immunocompromised hosts.

## Introduction

Coccidioidomycosis is a fungal infection caused by *Coccidioides immitis *and *Coccidioides posadasii *[1]. These fungi are endemic to the Southwestern United States, Mexico, and areas of South America. Initial infection takes place through the inhalation of aerosolized arthroconidia. The incidence of coccidioidomycosis has risen over recent decades in the United States, particularly in California, contributing to a growing clinical burden in endemic regions. While most infections are self-limited or subclinical, a minority of patients may develop disseminated disease. Those who come to clinical attention may present with upper respiratory infection-like symptoms, fever, arthralgias, and chest radiographic findings of hilar adenopathy with multiple infiltrates [2]. In a rare minority of infected patients, extrapulmonary symptoms may develop secondary to disseminated infection, with many of these symptoms manifesting months to years after initial infection [2].

Although dissemination may involve nearly any organ system, head and neck presentations are particularly rare and often underrecognized. Additionally, head and neck manifestations of coccidioidomycosis may be especially diagnostically challenging, as they can mimic malignancy or chronic inflammatory disease. There have been a few described cases of laryngeal involvement [3,4], upper airway mucosal lesions, and *Coccidioides* abscesses within the peritonsillar and retropharyngeal spaces [5]. Infection of the mastoid has also been reported, though this too remains a rare clinical entity [6]. Herein, we present the first documented case of simultaneous laryngeal and mastoid involvement and review the existing literature on otolaryngologic manifestations of disseminated coccidioidomycosis.

## Case presentation

We present the case of a 72-year-old Caucasian woman from the Southwestern United States with simultaneous laryngeal and mastoid involvement from coccidioidomycosis. She has been retired for several years and worked as a former accountant. She initially presented with progressive right ear pain, hearing loss, and hoarseness. She also reported a remote history of systemic symptoms, including fever, chills, and night sweats. Her medical history included gastric reflux, bronchiectasis status-post lobectomy from several decades prior, and Hashimoto’s thyroiditis. She had no immunosuppressive disorders and was not taking any immunosuppressive medications.

Her symptoms began with right-sided pulsatile tinnitus, gradually progressing over one year to peri-auricular pain and intermittent clear-yellow malodorous otorrhea. She initially presented to an outside facility and underwent balloon sinuplasty and right tympanostomy tube placement for presumed sinusitis. Her symptoms persisted. Several months later, she presented to our institution. Initial CT raised concern for tegmen dehiscence and CSF leak; however, repeat imaging revealed complete opacification of the right mastoid and middle ear with partial malleolar erosion without a definitive tegmen defect. Given persistent otologic symptoms, she was scheduled for a right tympanomastoidectomy.

Around the same time, she underwent a laryngological evaluation for progressive hoarseness prior to her scheduled tympanomastoidectomy surgery. Laryngoscopy revealed erythematous, edematous tissue with white patches involving the right true vocal fold, arytenoid, false vocal fold, and laryngeal surface of the epiglottis (Figure 1). CT neck showed asymmetry without a discrete mass or lymphadenopathy (Figure 2), while CT chest showed no evidence of pulmonary coccidioidomycosis. Microlaryngoscopy with biopsy showed spherules consistent with *Coccidioides*. Serology studies showed positive IgG and IgM via enzyme-linked immunosorbent assay, positive immunodiffusion reflex, and negative complement fixation.

**Figure 1:**
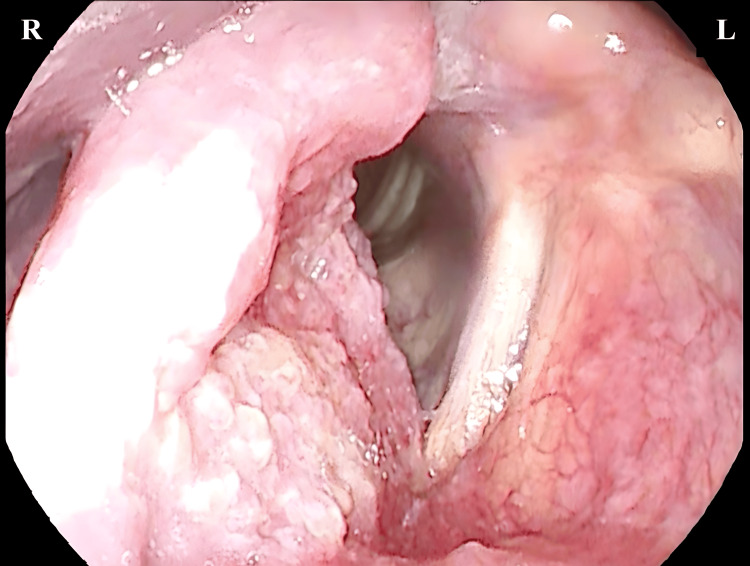
View of flexible laryngoscopy View of right (R) laryngeal pathology as visualized from flexible laryngoscopy. The pathology can be seen as erythematous, edematous tissue with white patches involving the right true vocal fold, right arytenoid cartilage, false vocal fold, and laryngeal surface of the epiglottis. These mucosal abnormalities prompted biopsy, which demonstrated spherules consistent with *Coccidioides*, establishing laryngeal involvement.

**Figure 2:**
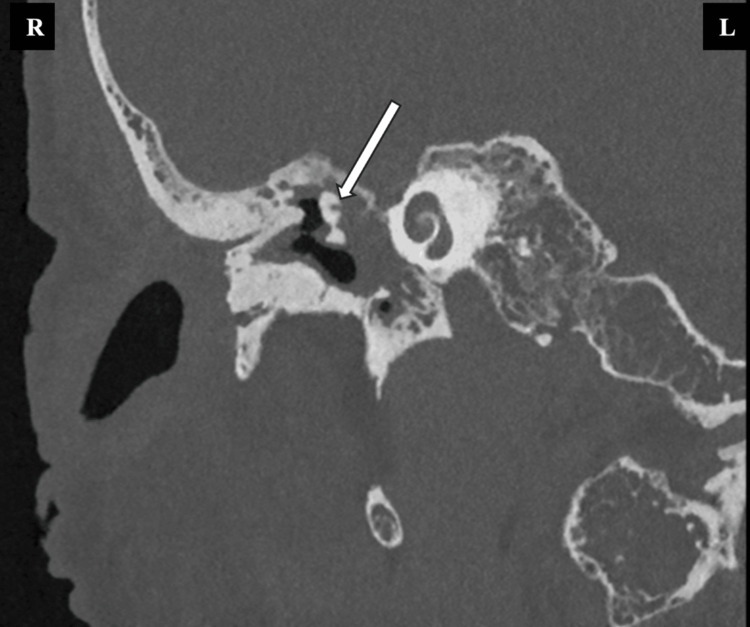
CT temporal bone scan Right CT temporal bone scan in a coronal view showing polypoid soft tissue along the inferior aspect of the external auditory canal, as well as near-complete opacification of the right middle ear and small malleolar head erosion (malleolar erosion depicted with arrow). These findings indicated a likely erosive otologic process and supported operative evaluation, with final pathology and cultures confirming *Coccidioides* mastoiditis.

The patient was started on fluconazole 800 mg daily by infectious disease (ID), consistent with the treatment guidelines for head and neck coccidioidomycosis per the Infectious Diseases Society of America guidelines [7]. Tympanomastoidectomy revealed polypoid granulation tissue throughout the middle ear and mastoid cavity, prompting additional exposure via a formal mastoidectomy with atticotomy. Operative pathology and cultures confirmed a diagnosis of *Coccidioides* mastoiditis. At her eight-week postoperative follow-up, the patient reported resolution of symptoms and continued to show clinical improvement.

Our manuscript involves the same patient described in the paper “Laryngeal coccidioidomycosis mimicking squamous cell carcinoma,” published in *Head and Neck Pathology *in February 2025 by Chuying Su and James S. Lewis Jr. However, we chose to pursue this manuscript because the patient’s clinical course was incomplete at the time of the earlier publication, and additional findings later emerged. Specifically, the patient showed simultaneous mastoid and laryngeal involvement of *Coccidioides*, a presentation that has not been previously reported in the literature and was not addressed in that report.

## Discussion

Coccidioidomycosis, endemic to the Southwestern United States, infrequently involves the head and neck. Among these uncommon cases, simultaneous laryngeal and mastoid involvement has not been previously described. Head and neck manifestations of coccidioidomycosis include cutaneous lesions, deep neck space abscesses, craniofacial bony destruction, and rare laryngeal involvement [7]. In one series, <1% of disseminated infections involved the head and neck [5]. *Coccidioides* may present as a chronic skin ulceration or subcutaneous abscess, with a predilection towards the central face [5]. Craniofacial abnormalities due to coccidioidomycosis may occur secondary to osteolytic changes. Galaviz-Aboytes et al. reported a rare case of an adult patient with bilateral periorbital nodules, which were found to be craniocutaneous coccidioidomycosis [8]. This patient was treated with surgical drainage followed by various azoles for three years; long-term outcomes were not reported.

Laryngeal disease has been described in a handful of cases, often mimicking malignancy on examination and imaging [9]. The most common presenting symptoms of laryngeal coccidioidomycosis are dysphonia or unsuspected airway compromise [10]. These classic characteristics are consistent with our patient’s presentation and symptomology. Converse et al. describe eleven cases of laryngeal coccidioidomycosis, and nearly all were secondary to primary *Coccidioides* infection elsewhere in the body [11]. Most patients received systemic antifungal therapy with fluconazole and reported improvement after an extended period of antifungal treatment (six weeks to greater than three years) (Table 1).

**Table 1:** Selected summary of previously reported head and neck coccidioidomycosis A selected summary of previously reported head and neck coccidioidomycosis cases, including anatomical site involvement, dissemination status, and treatment considerations.

Author	Year	Site	Dissemination status	Treatment
Lizárraga et al. [3]	2023	Larynx	No	N/A; patient passed away before antifungal management was started
Platt [4]	1977	Larynx	Yes	Amphotericin B for two years, followed by azoles
Arnold et al. [5]	2004	Various head and neck sites across 60 described cases (skull, facial bones, and cutaneous)	Yes	Majority of cases received amphotericin B; some cases also received azoles and/or surgical debridement
Schwartz et al. [6]	2023	Mastoid/otologic (nine cases)	No (for the majority of described cases)	Majority received azoles and/or surgical debridement; two received amphotericin B alone
Galaviz-Aboytes et al. [8]	2021	Craniocutaneous/craniofacial	No	Surgical drainage/biopsy; fluconazole 400 mg for three years
Boyle et al. [10]	1991	Larynx	Yes	Tracheostomy and fluconazole per experimental protocol
Converse et al. [11]	2020	Larynx (11 cases)	Yes (for the majority of described cases)	Fluconazole 800 mg; itraconazole 200 mg for various timeframes (six weeks to greater than three years)

Otologic involvement is similarly rare. Schwartz et al. described nine cases of otomastoiditis (Table 1) [6]. Eight of the nine affected patients lived in or had recently traveled to California. Most reported otalgia but did not have concurrent laryngeal or pulmonary coccidioidomycosis. Most were treated with fluconazole and experienced clinical improvement over several months. Our case thus adds to the literature by demonstrating discrete mastoid involvement in conjunction with laryngeal disease. Additionally, our patient’s coccidioidomycosis was restricted to the head and neck, and she did not show any signs of pulmonary coccidioidomycosis.

ID was consulted after tissue diagnosis confirmed laryngeal coccidioidomycosis. Due to the potential for skull base and central nervous system involvement from the mastoid, prolonged antifungal treatment with serial monitoring was recommended per the published guidelines for extrapulmonary/disseminated coccidioidomycosis. During workup, our patient was found to have mildly impaired kidney function. Therefore, fluconazole was initially started at a renally dosed 400 mg. However, once bony involvement was confirmed on operative pathology, this was switched to 800 mg dosing to reflect the greater severity of disease. ID attempted to transition her to a mold-active agent such as isavuconazonium sulfate, as mold-active agents offer the potential advantage of improved long-term disease suppression and are preferred for bony involvement/disseminated disease. However, the patient was continued on high-dose fluconazole due to tolerability and insurance accessibility.

Early recognition of head and neck coccidioidomycosis is essential to mitigate disease severity. The route of dissemination in this case remains incompletely understood. While contiguous spread via the upper respiratory tract is plausible, animal models suggest that hematogenous seeding may also localize to the middle ear [6]. Scalarone and Huntington also showed the localization of infection to the mastoid after intraperitoneal inoculation of *Coccidioides *in a murine model. These results suggest a potential non-contiguous route of hematogenous dissemination of *Coccidioides *to the middle ear [12]. Further research is required to better characterize the mechanism of *Coccidioides *infection within the head and neck region.

Our case illustrates the importance of including coccidioidomycosis in the differential diagnosis for chronic otologic or laryngeal complaints, especially in endemic regions. Travel and social history should be obtained from patients with symptoms such as new skin lesions, otalgia, hearing changes, or voice changes. Tissue biopsy, culture, and serology remain critical for diagnosis. Successful management often requires both surgical and medical approaches. Although surgical debridement may be initially performed to treat head and neck coccidioidomycosis, concurrent long-term antifungal therapy is required, although the optimal duration of antifungal therapy remains unknown [6].

## Conclusions

Coccidioidomycosis involving the head and neck is rare and diagnostically challenging. There are a few reported cases of coccidioidomycosis affecting the larynx or the mastoid individually. From our literature review, this is the first documented case of simultaneous involvement of both sites. Clinicians in endemic regions should maintain a high index of suspicion for *Coccidioides *infection in patients with persistent head and neck (e.g., otologic or laryngeal) symptoms that fail conventional therapy. Diagnosis relies on tissue biopsy with microbiologic confirmation, and management typically requires both surgical debridement and prolonged systemic antifungal therapy. Given the variability in clinical presentation and treatment response, individualized plans and close multidisciplinary coordination are essential to optimizing outcomes. Ultimately, this case highlights the importance of tissue diagnosis, antifungal therapy, and multidisciplinary coordination in the management of head and neck coccidioidomycosis.
